# Supplementary material for: Precisely spun super rotors
Source: Nat Commun. 2021 Apr 13;12:2201. doi: 10.1038/s41467-021-22342-6 (PMC8044131; doi:10.1038/s41467-021-22342-6)
Supplement: Supplementary file 1 — Supplementary Information [file 41467_2021_22342_MOESM1_ESM.docx]

**Section 1: Notation**

The rotational fine structure of the C^2^Π_1/2_ - X^2^Σ^+^ vibronic transitions (Fig. 1A) obeys electric dipole selection rules that allow change of total angular momentum Δ*J* = 0, ±1 and change of total parity “+” ↔ “-”(Fig. 2B). For the Hund’s case B ground X^2^Σ^+^ state *N* is a good number, determining parity and coarsely defining rotational level energies. Weak spin-rotation interaction results in a small splitting of the *N* levels into *J* = *N* ± ½ levels which have the same parity. The C^2^Π state is approximately Hund’s case A at low *J* and shifts towards Hund’s case B for high *J*. Each *J* level has a “+” and a “-” parity component split by a weak Λ-doubling interaction.

Although *N* is not a good quantum number for C^2^Π_1/2_, it is convenient to formulate selection rules in terms of change of *N*, defined as (*J*’ - ½) - *N*” for the C^2^Π_1/2_ and (*J*’ + ½) - *N*” for the C^2^Π_3/2_, where *J*’ refers to the C^2^Π level and *N*” refers to the X^2^Σ^+^ level. The allowed Δ*N* combinations result in four spectral branches: Δ*N* = -2 (^o^P_12_), Δ*N* = -1 (^p^Q_12_ and ^p^P_1_), Δ*N* = 0 (^q^R_12_ and ^q^Q_1_) and Δ*N* = +1 (^r^R_1_) for the C^2^Π_1/2_ - X^2^Σ^+^ transition. Similarly, Δ*N* = -1 (^p^P_2_), Δ*N* = 0 (^q^Q_2_ and ^q^P_21_), Δ*N* = +1 (^r^R_2_ and ^r^Q_21_) and Δ*N* = +2 (^s^R_21_) are allowed for the C^2^Π_3/2_ - X^2^Σ^+^. The fine structure in the doubly degenerate branches is determined by the spin-rotation splitting in the X^2^Σ^+^ state and cannot be resolved in our experiment, because of the lifetime broadening of the C^2^Π state. We use simplified labels for the rotational transitions with lowercase letters according to ΔN (o for -2, p for -1, q for 0, r for +1 and s for +2) followed by *N*” in parentheses.

The spectral lines were fitted to a ^2^Π - ^2^Σ^+^ transition expressions as defined in PGOPHER(*1*). The Hamiltonian for the X^2^Σ^+^ included perturbations by the A^2^Π state reported by Cameron et al(*2*). The fit results are reported in the Table 1, where *T_e_* is the electronic state origin, *ω_e_* and *ω_e_x_e_* are vibration frequency and anharmonicity, *B_e_*, *D_e_* and *A_e_* are rotational constant, centrifugal distortion and spin-orbit interaction, *α_e_*, *β_e_* and *α_A_* are their first-order vibrational expansion terms.


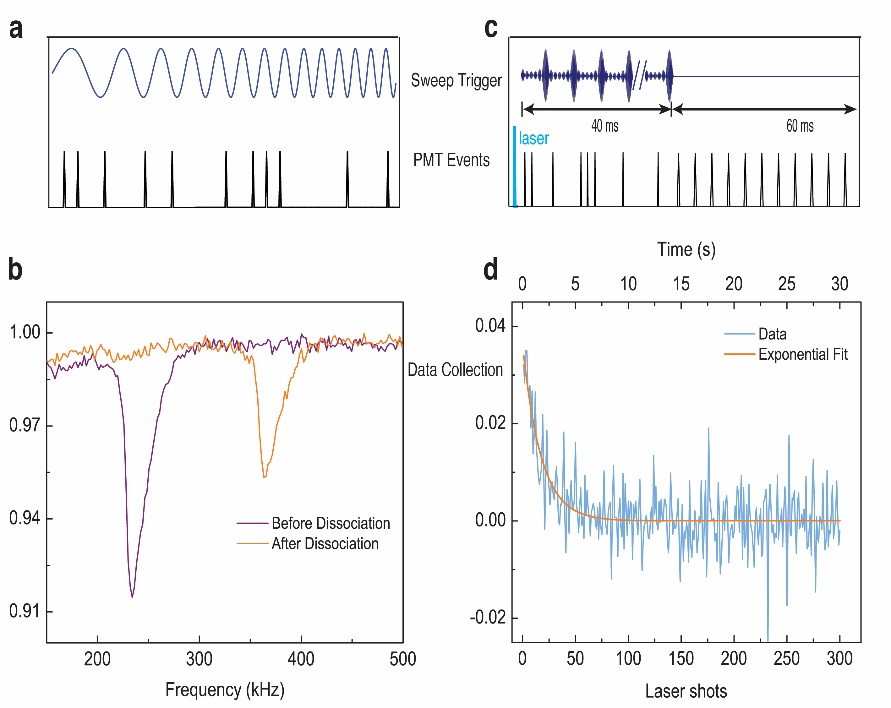


**Fig S1. LCFMS ion detection. (a)** Mass-spectrometer method waveform and PMT events. **(b)** *In-situ* mass spectra using this method. **(c)** Steady-state detection method wave form and PMT events. **(d)** Exponential decay of Ba+ fluorescence depletion in the steady-state method.

**Section 2: Super Rotor Dissociation for Probing Large Stretch Distances**

The observed predissociation could occur in principle in either the X or B state, both of which participate in optical pumping. Since the rate of optical pumping is much less than the fluorescence decay rate of the B state (~1000 s^-1^ vs 1.4·10^7^ s^-1^), molecules spend most of their time in X. The X,*v*=0 state however cannot dissociate even at *N*=230 for energetic reasons. The B state correlates with the excited Si^+^(^2^P) + O(^1^D) atomic asymptote and has enough energy to dissociate to ground state atoms (Si^+^(^2^P) + O(^3^P)) at *N*>140. Coupling of the B state to the ground state asymptote can occur via non-adiabatic interactions with 1^4^Σ^-^ and 1^4^Π excited electronic states(*3*)(Figs. 4, S2). The estimated upper-bound lifetimes of B, v = 0-3 super rotors due to non-adiabatic tunneling(*4*) to the 1^4^Σ^-^ and 1^4^Π states, based on reported spin-orbit coupling parameters(*3*) are shown in Fig. S2. These lifetime estimates only provide upper bounds because they do not account for the presently unknown inhomogeneous L-uncoupling perturbation. While weak for non-rotating molecules, this perturbation scales linearly with *N* and can dominate the electronic state coupling in super rotors. The B,*v* states become quasibound at some *N* and then become progressively more unstable at higher *N*. The observed rates of super rotor dissociation depend on the dissociation lifetime, the rate of optical pumping, and the radiative lifetime of the B state. The rates of 0.1-0.2 s^-1^ can be explained with 2.5-5 μs lifetime of the B,*v* which is consistent with our upper bound estimates for *N*=190,*v*=3 and *N*=220,*v*=1 super rotors. L-uncoupling may facilitate dissociation of lower vibrational levels in the super-rotors.

**
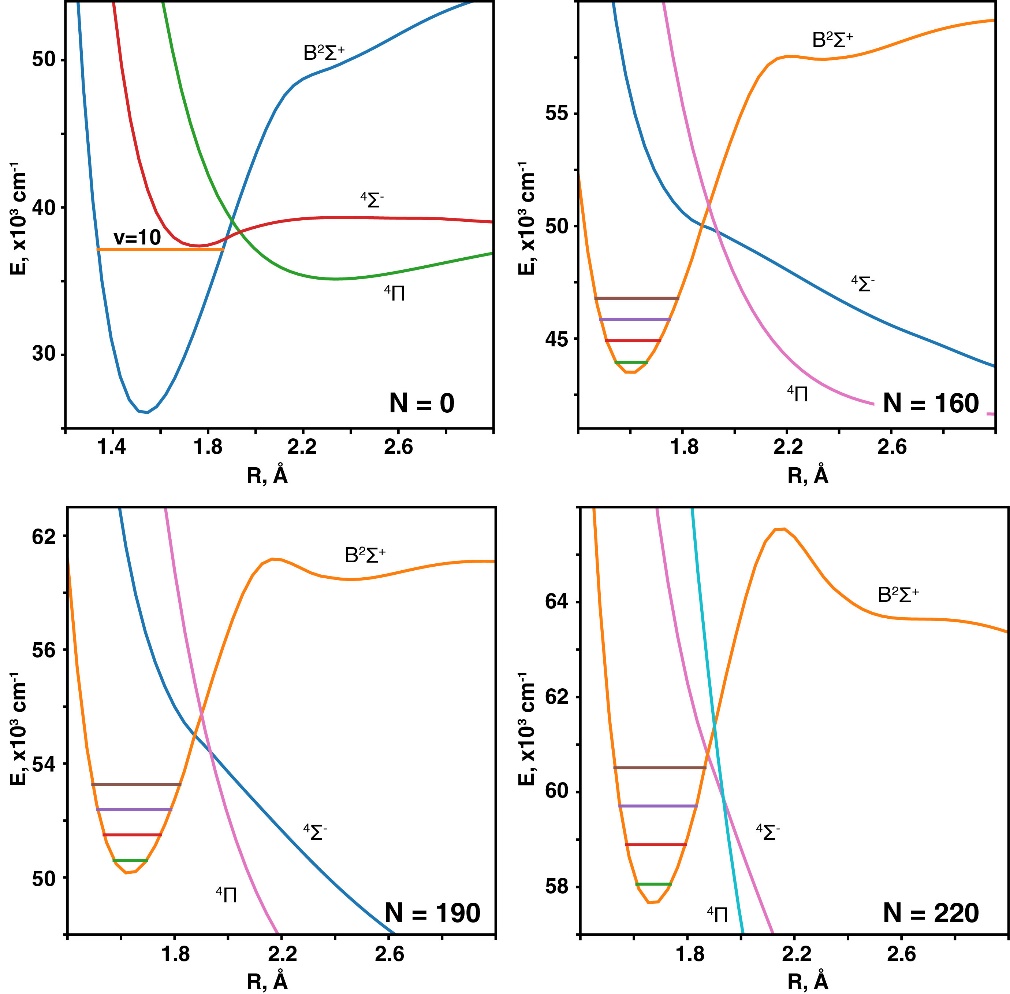
**

**Fig. S2.** **Potential energy curves of B^2^Σ^+^, 1^4^Σ^-^ and 1^4^Π states at various *N.*** In the *N*>0 panels, the lower bounds for their tunneling lifetimes in seconds are shown for *v*=0-3

The excited vibrational levels in the X state can be populated via off-diagonal decay of B-X transition which occurs with <3% probability. The X,*v*>0 states can undergo optical pumping to super rotors via the R-branch of diagonal B,v-X,v transitions (red Fortrat parabolas in Fig. 4) and off-diagonal decay to populate progressively higher vibrational states. However, vibrational excitation of the super rotors is limited by fast radiative decay of X,*v* to lower-lying A,*v* states. At *N*=0 this decay process is fast for X,*v*≥3 which is ~1000 cm-1 above the A,*v*=0 and decays to it with ~5 ms lifetime. The energy of the X,*v* state increases with N faster than the A,*v* states, and A,*v*=0 becomes the absolute ground vibronic state near *N*=145. Therefore it is likely that the vibrational population of the *N*>160 super rotors is constrained to the lowest X,*v* levels and observed dissociation occurs via excitation to B,*v*=0 or *v*=1.

The B-X spectrum is unknown past *N*=100 in the 0-0 band and for even lower rotational states in 1-1 and 2-2 bands(*2*). Therefore for preparation of these high *N* states, to determine the cut-off position of the spectral mask we relied on extrapolation of the spectroscopic constants of the B and X states augmented with our calculations of higher order centrifugal corrections *H* and *L*.

**Section 3: Super Rotor Spectroscopy for Probing Large Stretch Distances**

We have already discussed using dissociation measurements to probe molecular structure far from equilibrium geometry. Spectroscopy at high *N* can also be used to probe far from equilibrium geometry. We did not perform spectroscopy at *N*>67 because these energies are above the C^2^Π state dissociation threshold. However, choosing a different upper spectroscopy state would allow spectroscopic probing of X^2^Σ^+^ at much higher energies. Fig. S3 illustrates how measurement of energies and line widths even at low vibrational excitations of super rotor states can probe parts of the potential which would require very high vibrational excitation for *N*=0.


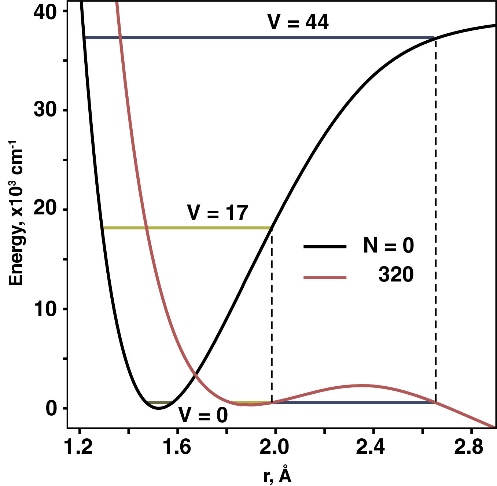


**Fig. S3. Spectroscopic probing of far from equilibrium geometry.** The SiO^+^ X^2^Σ^+^potential energy curves for the *N*=0 and *N*=320. For *N*=0, exciting to vibrational states *v*=17 and 44 are required to probe the same bond lengths probed at *v*=0 in the super rotor state.

**Section 4: Conditions for Steady-State Detection Method**

Assume that we have particle X with levels A and B connected by forward and reverse population transfer processes with first-order rate coefficients *k_f_* and *k_r_*. The density of X at level B is probed continuously as a function of time *t* via a destructive process, with first-order rate coefficient *k_p_*. Population flow is described by:

$$A\underset{\leftrightarrow}{k_{f},k_{r}}B\underset{\to}{k_{p}}dissociation$$

It can be shown that in the limiting case *k_f_* + *k_r_* >> *k_p_*, the measurement results in the exponential decay of number of X, with time constant proportional to the steady-state population of state B. Time-dependent populations of A and B, N_A_(t) and N_B_(t), can be found by solving a set of first order differential equations

$$\frac{dN_{A}}{dt}=-k_{f}N_{A}+k_{r}N_{B}$$

$$\frac{dN_{B}}{dt}=k_{f}N_{A}-\left( k_{r}+k_{p} \right)N_{B}$$

The set of equations can be solved by diagonalizing matrix of rate coefficients. The eigenvalues are

$$\lambda_{\pm}=\frac{-\left( k_{f}+k_{r}+k_{p} \right)\pm\sqrt{k_{p}^{2}+2\left( k_{r}-k_{f} \right)k_{p}+\left( k_{f}+k_{r} \right)^{2}}}{2}$$

When *k_f_* + *k_r_* >> *k_p_*,

$$\lambda_{-}\approx{-k}_{f}-k_{r}, \lambda_{+}\approx{-k}_{p}\frac{k_{f}}{k_{f}+k_{r}}\approx-k_{p}\frac{N_{B}}{N_{A}+N_{B}}$$

Here, $\lambda_{-}$ is the “fast” eigenvalue that describes equilibration of levels A and B, while $\lambda_{+}$ is the “slow” eigenvalue that describes dissociation of X.

Measurement of the $\lambda_{+}$ eigenvalue is equivalent to measurement of the product of population fraction in the B state and the rate of dissociation *k_p_*. If a pulsed laser is used, *k_p_* is a product of the repetition rate of the laser, e.g. 10 Hz, and a probability of dissociating molecules in the B state with a single laser pulse. The value of *k*_f_ + *k*_r_ is determined by optical pumping, and in our experiment is typically ~10 s^-1^. We lowered the laser fluence to ensure *k_f_* + *k_r_* >> *k_p_* and *k_p_* << 10 s^-1^ (so that we could measure an exponential population decay with the 10 Hz rep rate pulsed laser).

**Section 5: Theoretical Calculations**

First, a state-averaged complete active space self-consistent field (SA-CASSCF(*5, 6*) with a full valence active space that distributes 11 electrons in eight active space orbitals (silicon 3s3p and oxygen 2s2p orbitals) was performed. Dynamical correlation was included by single and double excitation of the SA-CASSCF reference wavefunction using the internally contracted multireference configuration interaction (icMRCISD) corrected by Davidson's method (*7, 8*). For atomic basis sets, the aug-cc-pwCV5Z sets were employed(*9, 10*). To account for the spin-orbit coupling, the spin-orbit matrix elements were calculated using the Breit-Pauli operator(*11*) at the CASSCF/aug-cc-pwCV5Z level of theory. All electronic structure calculations were performed using the MOLPRO program package(*12, 13*). Energies of bound and quasibound rovibrational states and tunneling widths/lifetimes for quasibound states were calculated using the program Level(*14*). The effect of the spin-orbit coupling on the quasibound states was estimated by the inclusion of the spin-orbit terms to the non-relativistic Hamiltonian using the program Duo(*15*). Couplings of the C^2^Π electronic state with 1^4^Π, 2^2^∆, and 2^2^Σ^-^ were considered. The resonance energy and width of quasibound states was estimated by the calculation of the averaged density of states using the stabilization method(*16*). These results are shown in Table S1.

**Section 6: Extensions of the Optical Pumping Technique**

Our optical pumping scheme relied on favorable overlap between vibrational levels of X and B states of SiO^+^. Molecules with less favorable overlap can be optically pumped if a sufficient number of vibrational levels in the ground electronic state are repumped. Technical improvements to enhance bandwidth, resolution and temporal control of the optical pumping laser have been suggested(*17*). They can be used to greatly improve the rotational state purity and might allow optical pumping of many different species. The only strict requirement for quantum state control by optical pumping is that the molecule has an excited state which radiatively decays to the target state much faster than radiative or non-radiative decay processes deplete the target state.

Although we control the absolute magnitude of the super-rotor angular momentum, we have not yet attempted to orient the axis of rotation in the lab frame. (It should be noted that some degree of control of the axis of rotation is a natural outcome of the optical centrifuge technique). It was suggested(*18*) that by using circularly polarized light for the R-branch pumping, a high degree of orientation of rotation can be achieved. Optical pumping using a combination of circular polarization on the R-branch and linear polarization on the P-branch can pump population to the single highest magnetic sublevel in a desired rotational state.


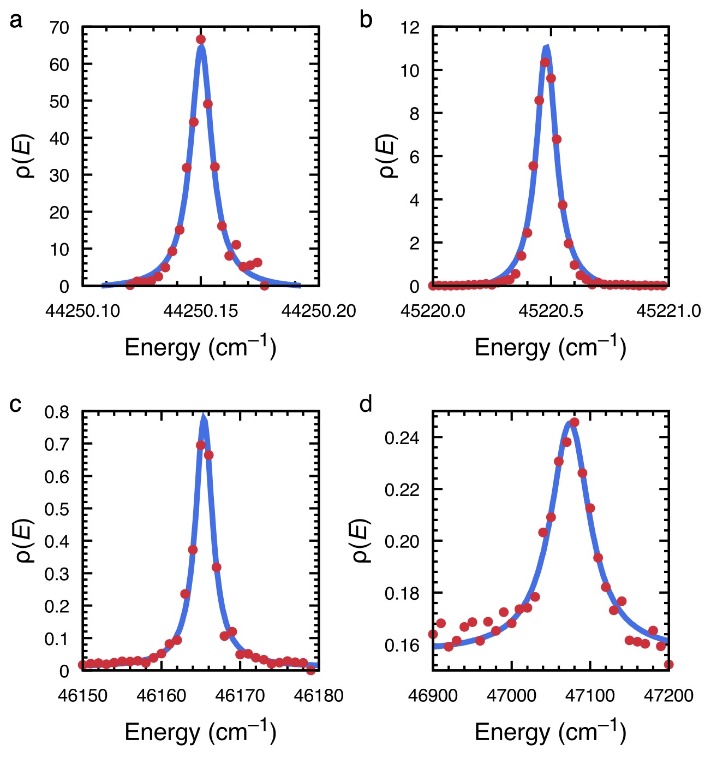


**Fig S4. Quasibound C^2^Π states.** Calculated averaged density of states shows quasibound resonances corresponding to **(a)** *v*=0, **(b)** *v*=1, **(c)** *v*=2, and **(d)** *v*=3.

| *v* | *G*(*v*) (cm^–1^) | Γ (cm^–1^) | τ (s) |
| --- | --- | --- | --- |
| 0 | 44250.1 | 0.0110 | 4.84 × 10^–10^ |
| 1 | 45220.5 | 0.1021 | 5.20 × 10^–11^ |
| 2 | 46165.4 | 2.640 | 2.01 × 10^–12^ |
| 3 | 47074.4 | 60.51 | 8.77 × 10^–14^ |

**Table S1. Calculated SiO^+^ C^2^Π quasibound state properties.** Vibrational energies, *G*(*v*), with respect to the electronic ground state, resonance widths (Γ), and lifetimes (τ).

***v*=0, Ω = 0.5**

**o**

| E | N | Sample |
| --- | --- | --- |
| 44024.60 | 8 | N=10 |
| 44021.69 | 9 | N=10 |
| 44018.64 | 10 | N=10 |
| 44015.41 | 11 | N=10 |

**p**

| 44034.53 | 8 | N=10 |
| --- | --- | --- |
| 44032.83 | 9 | N=10 |
| 44031.29 | 10 | N=10 |
| 44029.40 | 11 | N=10 |

**q**

| 44044.66 | 0 | N=0 |
| --- | --- | --- |
| 44045.10 | 1 | N=0 |
| 44029.52 | 23 | N=25 |
| 44027.75 | 24 | N=25 |
| 44025.68 | 25 | N=25 |
| 44023.57 | 26 | N=25 |

**r**

| 44046.56 | 0 | N=0 |
| --- | --- | --- |
| 44048.38 | 1 | N=0 |
| 44058.61 | 8 | N=10 |
| 44059.42 | 9 | N=10 |
| 44060.38 | 10 | N=10 |
| 44061.18 | 11 | N=10 |
| 44061.50 | 24 | N=25 |
| 44060.72 | 25 | N=25 |
| 44059.94 | 26 | N=25 |
| 44043.38 | 37 | N=40 |
| 44040.70 | 38 | N=40 |
| 44038.23 | 39 | N=40 |
| 44035.92 | 40 | N=40 |
| 44033.46 | 41 | N=40 |
| 44031.34 | 42 | N=40 |

***v*=0, Ω = 1.5**

**p**

| 44073.65 | 38 | N=40 |
| --- | --- | --- |
| 44067.79 | 39 | N=40 |
| 44061.99 | 40 | N=40 |
| 44055.81 | 41 | N=40 |
| 44049.58 | 42 | N=40 |
| 44043.38 | 43 | N=40 |

**q**

| 44038.30 | 54 | N=55 |
| --- | --- | --- |
| 44031.96 | 55 | N=55 |
| 44025.64 | 56 | N=55 |
| 44019.20 | 57 | N=55 |
| 44012.77 | 58 | N=55 |
| 44006.01 | 59 | N=55 |

**r**

| 44223.72 | 1 | N=0 |
| --- | --- | --- |
| 44224.24 | 2 | N=0 |
| 44049.85 | 65 | N=67 |
| 44043.94 | 66 | N=67 |
| 44037.76 | 67 | N=67 |
| 44031.39 | 68 | N=67 |
| 44025.01 | 69 | N=67 |
| 44018.27 | 70 | N=67 |

**s**

| 44225.20 | 0 | N=0 |
| --- | --- | --- |

***v*=1, Ω = 0.5**

**o**

| 44987.28 | 7 | N=10 |
| --- | --- | --- |
| 44984.53 | 8 | N=10 |
| 44981.26 | 9 | N=10 |
| 44978.02 | 10 | N=10 |
| 44974.71 | 11 | N=10 |

**p**

| 44995.96 | 7 | N=10 |
| --- | --- | --- |
| 44994.43 | 8 | N=10 |
| 44992.28 | 9 | N=10 |
| 44990.65 | 10 | N=10 |
| 44988.32 | 11 | N=10 |

**q**

| 45004.61 | 0 | N=0 |
| --- | --- | --- |
| 45005.03 | 1 | N=0 |

**r**

| 45006.88 | 0 | N=0 |
| --- | --- | --- |

***v*=1, Ω = 1.5**

**p**

| 45163.87 | 8 | N=10 |
| --- | --- | --- |
| 45160.87 | 9 | N=10 |
| 45157.75 | 10 | N=10 |
| 45154.41 | 11 | N=10 |

**q**

| 45173.6 | 8 | N=10 |
| --- | --- | --- |
| 45171.95 | 9 | N=10 |
| 45170.22 | 10 | N=10 |
| 45168.3 | 11 | N=10 |

**r**

| 45103.38 | 43 | N=45 |
| --- | --- | --- |
| 45099.41 | 44 | N=45 |
| 45094.51 | 45 | N=45 |
| 45090.03 | 46 | N=45 |
| 45085.25 | 47 | N=45 |
| 45080.38 | 48 | N=45 |
| 45075.05 | 49 | N=45 |
| 45054.1 | 53 | N=55 |
| 45048.42 | 54 | N=55 |
| 45042.54 | 55 | N=55 |
| 45036.47 | 56 | N=55 |
| 45030.44 | 57 | N=55 |
| 45024.41 | 58 | N=55 |
| 45011.31 | 60 | N=62 |
| 45005.3 | 61 | N=62 |
| 44998.77 | 62 | N=62 |
| 44991.83 | 63 | N=62 |
| 44984.65 | 64 | N=62 |

**s**

| 45185.78 | 0 | N=0 |
| --- | --- | --- |
| 45086.98 | 61 | N=62 |
| 45081.25 | 62 | N=62 |
| 45075.76 | 63 | N=62 |
| 45070.25 | 64 | N=62 |
| 45064.32 | 65 | N=62 |

***v*=2, Ω = 0.5**

**q**

| 45942.27 | 0 | N=0 |
| --- | --- | --- |

**r**

| 45944.24 | 0 | N=0 |
| --- | --- | --- |

***v*=2, Ω = 1.5**

**s**

| 46123.93 | 0 | N=0 |
| --- | --- | --- |

**Table S2.** **C-X line list.** Line values are extracted from the spectra.

**References**

1. C. M. Western, PGOPHER: A program for simulating rotational, vibrational and electronic spectra. *Journal of Quantitative Spectroscopy and Radiative Transfer* **186**, 221-242 (2017).

2. S. D. Rosner, R. Cameron, T. J. Scholl, R. A. Holt, A study of the X-2 Sigma(+) and A(2)Pi states of SiO+ using fast-ion-beam laser spectroscopy. *Journal of Molecular Spectroscopy* **189**, 83-94 (1998).

3. R. Li *et al.*, Laser cooling of the SiO+ molecular ion: A theoretical contribution. *Chemical Physics* **525**, 110412 (2019).

4. C. Zhu, Y. Teranishi, H. Nakamura, in *Advances in Chemical Physics*. pp. 127-233.

5. P. J. Knowles, H.-J. Werner, An efficient second-order MC SCF method for long configuration expansions. *Chem. Phys. Lett.* **115**, 259-267 (1985).

6. H. J. Werner, P. J. Knowles, A Second Order {MCSCF} Method with Optimum Convergence. *J. Chem. Phys.* **82**, 5053-5053 (1985).

7. P. J. Knowles, H.-J. Werner, An efficient method for the evaluation of coupling coefficients in configuration interaction calculations. *Chem. Phys. Lett.* **145**, 514-522 (1988).

8. H.-J. Werner, P. J. Knowles, An efficient internally contracted multiconfiguration–reference configuration interaction method. *J. Chem. Phys.* **89**, 5803-5814 (1988).

9. R. A. Kendall, J. T. H. Dunning, R. J. Harrison, Electron Affinities of the First-Row Atoms Revisited. Systematic Basis Sets and Wave Functions. *J. Chem. Phys.* **96**, 6796-6806 (1992).

10. K. A. Peterson, J. T. H. Dunning, Accurate correlation consistent basis sets for molecular core–valence correlation effects: The second row atoms Al–Ar, and the first row atoms B–Ne revisited. *J. Chem. Phys.* **117**, 10548-10560 (2002).

11. A. Berning, M. Schweizer, H.-J. Werner, P. J. Knowles, P. Palmieri, Spin-orbit matrix elements for internally contracted multireference configuration interaction wavefunctions. *Mol. Phys.* **98**, 1823-1833 (2000).

12. H. J. Werner, P. J. Knowles, G. Knizia, F. R. Manby, M. Schütz, Molpro: a general-purpose quantum chemistry program package. *WIREs Comput Mol Sci* **2**, 242-253 (2012).

13. H. J. Werner *et al.* (Cardiff, UK, 2015).

14. R. J. Le Roy, LEVEL: A computer program for solving the radial Schrödinger equation for bound and quasibound levels. *J. Quant. Spectrosc. Radiat. Transf.* **186**, 167-178 (2017).

15. S. N. Yurchenko, L. Lodi, J. Tennyson, A. V. Stolyarov, Duo: A general program for calculating spectra of diatomic molecules. *Comput. Phys. Commun.* **202**, 262-275 (2016).

16. V. A. Mandelshtam, T. R. Ravuri, H. S. Taylor, Calculation of the density of resonance states using the stabilization method. *Phys. Rev. Lett.* **70**, 1932-1935 (1993).

17. P. R. Stollenwerk, I. O. Antonov, S. Venkataramanababu, Y.-W. Lin, B. C. Odom, Cooling of a Zero-Nuclear-Spin Molecular Ion to a Selected Rotational State. *Phys. Rev. Lett.* **125**, 113201 (2020).

18. J. Li, J. T. Bahns, W. C. Stwalley, Scheme for state-selective formation of highly rotationally excited diatomic molecules. *The Journal of Chemical Physics* **112**, 6255-6261 (2000).
